# The Need for Primary Care Beyond the Postnatal Period: Uncontrolled Hypertension-Induced Thrombotic Microangiopathy in a 32-Year-Old With a History of Preeclampsia Presenting Three Months Postpartum

**DOI:** 10.7759/cureus.35582

**Published:** 2023-02-28

**Authors:** Elena N Zamora, Justin A Riojas, Kristen L Robinson, Shira K Goldstein

**Affiliations:** 1 Family and Community Medicine, University of Texas Health Science Center at Houston, Houston, USA; 2 Family and Community Medicine, University of Texas at Houston, Houston, USA

**Keywords:** preeclampsia, thrombotic microangiopathy, pregnancy-induced thrombocytopenia, pregnancy-induced, thrombocytopenia, hellp syndrome, hypertensive urgency, microangiopathic hemolytic anemia

## Abstract

A 32-year-old African American female with a past medical history of uncontrolled hypertension and preeclampsia with severe features presented to the emergency department with three days of shortness of breath, chest pain, bloody cough, and non-bloody diarrhea without any known prior viral syndrome. On presentation, she was diagnosed with a hypertensive emergency with renal and cardiac dysfunction. Laboratory workup revealed leukocytosis, normocytic anemia, and thrombocytopenia. The remainder of the laboratory data were significant for hemolysis. Differential diagnosis included thrombotic thrombocytopenic purpura (TTP)/hemolytic uremic syndrome (HUS); therefore, the patient was initiated on TTP treatment of pulsed dose steroids and plasma exchange. However, once the ADAMTS13 test returned negative, plasma exchange was stopped and the patient’s profile returned to normal with supportive care and aggressive blood pressure control, as she had hypertension-induced thrombotic microangiopathy.

## Introduction

Thrombotic microangiopathy (TMA) is a condition characterized by the triad of microangiopathic hemolytic anemia, thrombocytopenia, and end-organ damage. Microcirculatory lesions cause schistocyte formation and platelet consumption. Characteristic laboratory findings of TMA include thrombocytopenia, elevated lactate dehydrogenase, and hemolytic anemia. Causes of TMA include hemolytic uremic syndrome (HUS), thrombotic thrombocytopenic purpura (TTP), prescribed and unprescribed medications, and severe hypertension, most commonly with systolic blood pressures above 200 mmHg. Here, we report a case of hypertension-induced TMA in a patient with a history of preeclampsia with severe features who was not on optimal anti-hypertensive management.

## Case presentation

A 32-year-old African American female with a history of preeclampsia with severe features complicated by chronic hypertension (one year prior) presented to the emergency department with three days of shortness of breath, chest pain, bloody cough, and non-bloody diarrhea. She had been non-adherent to her antihypertensive medications of labetalol and lisinopril for an unknown duration. On presentation, the patient's temperature was 98.4°F, blood pressure was 225/145 mmHg, heart rate was 101 beats per minute, and oxygen saturation was 80% on room air. Labs were significant for serum creatinine at 2.1 (baseline: 0.6), elevated troponins at 1.8 (normal range: 0.00 to 0.04 ng/ml), white blood cell count of 18.1 × 109/L (normal range: 4.5-11.0 × 109/L), acute anemia with hemoglobin at 10.7 gm/dL (normal range: 13.5-17.5 gm/dL), low platelet count at 53 × 109/L (150-400 × 109/L), elevated reticulocyte count at 5.4%, normal total bilirubin at 0.6 mg/dL (0.1-1.2 mg/dL), elevated lactate dehydrogenase at 826 U/L (140-280 U/L), low haptoglobin < 8 mg/dL, and a negative direct Coombs test. A peripheral blood smear was significant for six schistocytes/hpf (2.6%).

The patient was initially treated for hypertensive emergency and immune thrombocytopenic purpura (ITP)/TTP with dexamethasone. Hematology was consulted for concern of TTP/HUS vs. hypertension-induced TMA. TTP was initially highest on the differential diagnoses as her PLASMIC score was 5. Therefore, she was treated with methylprednisolone 125 mg every six hours and three rounds of plasma exchange. However, as her ADAMTS13 level on the sample collected prior to therapeutic plasma exchange (TPE) was 57%, TTP was ruled out. Therefore, all treatments were stopped, and after five days, the patient’s blood pressure and platelets returned to normal, with a discharge platelet count of 279,000.

## Discussion

Management of hypertension in pregnant vs. non-pregnant population

In the general population, the choice of hypertension medication should aim to reduce complications such as stroke, myocardial infarction, and mortality. Optimizing blood pressure control helps to reduce the risk of end-organ damage. Women with gestational hypertension and preeclampsia have a three-fold and six-fold increased rate of future hypertension, respectively. In patients with pregnancy-induced hypertension, end-organ damage typically presents as hemolysis, thrombocytopenia, or transaminitis, a triad known as hemolysis, elevated liver enzymes, and low platelet count (HELLP) syndrome [1]. Complications of HELLP syndrome include placental abruption, kidney failure, and disseminated intravascular coagulation (DIC). During pregnancy, non-selective beta blocker labetalol and the calcium channel blocker (CCB) nifedipine are generally used due to good side effect profiles. Methyldopa is not as effective but is considered a second-line therapy after CCBs and beta blockers. In breastfeeding patients, anti-hypertensives that are not excreted into breast milk are the treatment of choice.

Because the management of hypertension during pregnancy differs from that of hypertension in the non-pregnant population, postpartum patients who remain hypertensive should continue to follow up with their primary care physician for blood pressure management. Evidence showing thiazides improve blood pressure outcomes makes this class of medications the first-line drug for most patients with hypertension. The optimal time to transition from medications used during gestational hypertension to that according to recommendations for the general population is during the postpartum period after breastfeeding has been stopped.

Pathogenesis of hypertension-induced TMA and HELLP syndrome

Multiple cases of malignant hypertension-induced TMA have been reported. Although the exact pathogenesis is not confirmed, the literature suggests erythrocyte fragmentation passing through narrowed vasculature damages the endothelium causing activation of the coagulation cascade leading to fibrinoid necrosis, edema of arterioles, and local platelet aggregation [2]. Thus, patients with hypertension-induced TMA will present with hemolysis (microangiopathic hemolytic anemia), thrombocytopenia, and end-organ damage (such as renal failure).

Like the proposed pathogenesis for hypertension-induced TMA, HELLP syndrome is believed to be caused by endothelial cell injury triggering a coagulation cascade, which is triggered by the release of von Willebrand factor (vWF). Increased exposure of platelets to vWF results in thrombocytopenia. The coagulation cascade leads to erythrocyte fragmentation and the release of fibrin, which build up in the liver causing vascular congestion and hepatic damage, and inflammatory cytokines released by the placenta potentiate the cascade [3]. Thus, patients with HELLP syndrome will present with hemolysis, thrombocytopenia, and end-organ liver damage.

## Conclusions

Both hypertension-induced TMA and HELLP syndrome present with microangiopathic hemolytic anemia and thrombocytopenia. Both pathogeneses of TMA and HELLP syndrome involve erythrocyte damage causing coagulation cascade and platelet destruction. In pregnancy, non-selective beta-blockers and CCBs are preferred, whereas thiazides are first-line drugs for most non-pregnant patients with hypertension. Clinicians should ensure adequate blood pressure control is maintained during pregnancy and postpartum to prevent HELLP syndrome and hypertension-induced TMA, respectively. Patients should be transitioned from obstetrician management of their hypertension to primary care management beyond the postpartum period.
